# 恶性胸膜间皮瘤的内科治疗研究进展

**DOI:** 10.3779/j.issn.1009-3419.2025.102.18

**Published:** 2025-05-20

**Authors:** Jie YANG, Fanming KONG

**Affiliations:** ^1^300381 天津，天津中医药大学第一附属医院肿瘤科; ^1^Department of Oncology, First Teaching Hospital of Tianjin University of Traditional Chinese Medicine; ^2^国家中医针灸临床医学研究中心; ^2^National Clinical Research Center for Chinese Medicine Acupuncture and Moxibustion; ^3^天津市中医肿瘤研究所; ^3^Tianjin Cancer Institute of Traditional Chinese Medicine,Tianjin 300381, China

**Keywords:** 恶性胸膜间皮瘤, 分子靶向治疗, 免疫治疗, 化疗, 联合疗法, Malignant pleural mesothelioma, Molecular targeted therapy, Immunotherapy, Chemotherapy, Combination therapy

## Abstract

恶性胸膜间皮瘤（malignant pleural mesothelioma, MPM）是一种源自胸膜间皮细胞的高度侵袭性恶性肿瘤，主要与石棉暴露相关，且预后通常较差。由于该病早期缺乏特异性临床表现，诊断具有一定挑战性，导致多数患者确诊时已处于晚期，从而限制了外科手术治疗的效果，因此需要依赖全身治疗。尽管培美曲塞联合铂类化疗仍为不可切除MPM的一线标准治疗方案，但其疗效有限，亟需更有效的治疗策略。近年来，双免疫检查点抑制剂在MPM治疗中取得了重要进展，显著改善了患者的生存预后。随着对MPM分子生物学研究的深入，靶向治疗为患者提供了潜在的个性化治疗选择。此外，细胞治疗、溶瘤病毒疗法等新型治疗策略的治疗潜力已开始显现。本文综述了MPM的最新内科治疗进展，并对未来治疗方向进行展望，以期为临床实践提供参考。

## Abstract (en)

Malignant pleural mesothelioma (MPM) is a highly aggressive malignancy originating from mesothelial cells of the pleura, primarily associated with asbestos exposure, and is often characterized by poor prognosis. Due to the lack of specific clinical manifestations in the early stages, the diagnosis of MPM presents a significant challenge, leading to most patients being diagnosed at an advanced stage, which limits the effectiveness of surgical treatment. Consequently, systemic therapies are commonly required. Although Pemetrexed in combination with Platinum-based chemotherapy remains the first-line standard treatment for unresectable MPM, its therapeutic efficacy is limited, and more effective treatment strategies are urgently needed. In recent years, immune checkpoint inhibitors have made significant progress in the treatment of MPM, markedly improving patient survival outcomes. With the increasing depth of molecular biological research on MPM, targeted therapies offer promising personalized treatment options. Additionally, the therapeutic potential of novel strategies such as cell therapy and oncolytic virus therapy is beginning to emerge. This review summarizes the latest advancements in the medical treatment of MPM and looks forward to future therapeutic directions, aiming to provide insights for clinical practice.

恶性胸膜间皮瘤（malignant pleural mesothelioma, MPM）是一种起源于胸膜间皮细胞的恶性肿瘤，表现出高度侵袭性和致命性。根据2024年的GLOBOCAN数据^[1]^显示，2022年全球恶性间皮瘤新发病例为30,618例，死亡25,372例，病死率较高。恶性间皮瘤的预后较差，中位总生存期（median overall survival, mOS）为8-12个月^[2]^。研究^[3]^证实，MPM发生与职业性石棉暴露密切相关，在男性中约85%的病例可归因于此，其他已知危险因素包括电离辐射和基因突变。该病通常发生在中老年人中，诊断的中位年龄为76岁^[4]^。MPM包括上皮样型（约占2/3）、肉瘤样型和双相型三种病理类型，不同亚型的预后差异显著。接受手术治疗后，上皮样型患者的mOS可达19个月，双相型为12个月，而肉瘤样型仅为4个月^[5]^。因此，手术治疗目前主要推荐用于上皮样型MPM。然而，是否应对非上皮型MPM患者实施手术治疗仍存在争议。美国国立综合癌症网络（National Comprehensive Cancer Network, NCCN）指南指出，对于肉瘤样型或双相型组织学的患者，仅应在早期、肿瘤负荷较小且无淋巴结受累的情况下才考虑手术。因此，对于经过严格筛选的双相型或肉瘤样型患者，手术治疗可能仍具有一定的潜力^[6]^。

目前，尚无大型随机临床试验直接比较MPM患者手术与非手术治疗的疗效。对于不可切除的MPM患者，治疗选择仍然有限。NCCN指南^[7]^推荐培美曲塞联合铂类化疗作为一线治疗方案。MAPS研究^[8]^显示，联合抗肿瘤血管生成靶向药物贝伐珠单抗可进一步延长患者生存期。随着对MPM分子机制理解的深入，靶向治疗的研究不断推进，未来有望为患者带来更加精准的治疗策略。免疫治疗方面，CheckMate 743研究^[9]^证实伊匹木单抗联合纳武利尤单抗显著延长了不可切除MPM患者的生存期，优于传统化疗，已被NCCN指南和《中国恶性胸膜间皮瘤临床诊治全国专家共识》推荐为一线治疗方案，特别适用于肉瘤样型MPM^[7,10]^。关于MPM治疗领域的关键临床研究，我们在表1中进行了系统汇总^[8,9,11-16]^。此外，细胞治疗、溶瘤病毒疗法及其他新兴免疫治疗策略的研究亦在积极推进中。该疾病的复杂性和异质性为联合治疗提供成功的机会。本文将综述MPM内科治疗的最新研究进展，并探讨未来有前景的治疗策略。

**表 1:** MPM治疗领域关键临床试验设计与结果总结

Study	Study design	Population	Intervention	Primary endpoint	Key findings
MAPS^[8]^	Phase III, randomized, open-label	Unresectable MPM	Pemetrexed+Cisplatin+Bevacizumab *vs* Pemetrexed+Cisplatin	OS	OS: 18.8 (triplet) vs 16.1 mon (doublet)
CheckMate 743^[9]^	Phase III, randomized, open-label	Unresectable MPM	Nivolumab+Ipilimumab *vs* Pemetrexed+Cisplatin/Carboplatin	OS	mOS: 18.1 (IO) *vs* 14.1 mon (chemotherapy)
BEAT-Meso^[11]^	Phase III, multicenter, randomized, open-label	Advanced MPM	Atezolizumab+Bevacizumab+ chemotherapy *vs* Bevacizumab+ chemotherapy	OS	mOS: 20.5 (combination) *vs* 18.1 mon (control); Non-epithelioid histology: HR for OS=0.51
ATOMIC-Meso^[12]^	Phase II/III, randomized, double-blind	Non-epithelioid MPM	ADI-PEG 20+Pemetrexed+Cisplatin *vs* placebo+Pemetrexed+Cisplatin	OS	mOS: 9.3 (experimental) *vs* 7.7 mon (control)
CONFIRM^[13]^	Phase III, multicenter, randomized, double-blind, placebo-controlled	Patients with malignant pleural or peritoneal mesothelioma progressed after Platinum-based chemotherapy	Nivolumab *vs* placebo	OS, PFS	mOS: 10.2 *vs* 6.9 mon; mPFS: 3.0 *vs* 1.8 mon
DREAM^[14]^	Phase II, multicenter, single-arm, open-label	Treatment-naive unresectable MPM	Durvalumab+Platinum+Pemetrexed	6-mon PFS rate	6-mon PFS rate: 57%
PrE0505^[15]^	Phase II, single-arm, multicenter	Treatment-naive unresectable MPM	Durvalumab+Platinum+ Pemetrexed	OS	mOS: 20.4 mon
IND227^[16]^	Phase III, randomized, open-label	Treatment-naive advanced MPM	Pembrolizumab+Platinum+Pemetrexed *vs* Platinum+Pemetrexed	OS	Non-epithelioid subgroup: mOS: 12.3 (combination) *vs* 8.2 mon (chemotherapy)

MPM: malignant pleural mesothelioma; OS: overall survival; PFS: progression-free survival; mOS: median OS; IO: immuno-oncology.

## 1 手术可切除MPM患者的围手术期全身治疗

在可切除的I-IIIA期MPM患者中，新辅助或辅助顺铂（或卡铂）联合培美曲塞治疗为标准方案^[7]^。考虑到手术过程中出血的风险，通常不推荐在新辅助治疗中联合贝伐珠单抗。近年来，围手术期治疗研究聚焦于免疫治疗及其与化疗的联合策略。SWOG1619试验^[17]^旨在评估新辅助治疗方案阿替利珠单抗联合顺铂加培美曲塞在可切除上皮型或双相型MPM患者中的安全性。25例患者接受了新辅助化疗和免疫治疗后，18例病情稳定（stable disease, SD）或部分缓解（partial response, PR），并接受了胸膜切除术/剥脱术（pleurectomy/decortication, P/D）或胸膜外全肺切除术（extrapleural pneumonectomy, EPP）。术后15例继续维持阿替利珠单抗治疗。研究未见≥3级的治疗相关不良事件（treatment-related adverse events, TRAEs）。尽管疗效评估和长期毒性的结果尚待进一步报告，但初步数据表明该治疗方案具有良好的安全性。一项单臂临床试验^[18]^（NCT04162015）结果显示，新辅助纳武利尤单抗联合化疗或对MPM患者P/D术后产生积极的预后影响（mOS达41.0个月）。此外，另一项临床试验（NCT03760575）正在评估帕博利珠单抗联合化疗应用于可手术切除MPM患者的可行性。

II期临床试验（NCT02592551）^[19]^评估了双免疫检查点抑制剂（immune checkpoint inhibitors, ICIs）在可切除MPM患者中的疗效。该试验中，患者接受了度伐利尤单抗单药或联合替西木单抗新辅助治疗。研究发现两种治疗方案均能促进CD8^+^ T细胞在肿瘤中的浸润，联合治疗组能诱导CD57^+^效应记忆T细胞群从骨髓动员到血液循环。中位随访34.1个月时，单药组mOS为14.0个月，联合治疗组尚未达到，提示其可能带来更长生存期。然而，联合治疗显示出27%的3级及以上免疫相关AEs（immune-related AEs, irAEs）发生率明显高于单药组的8%。另一项研究（NCT03918252）正在评估纳武利尤单抗联合伊匹木单抗的新辅助治疗，以进一步明确双ICIs在围手术期的应用前景。总体来看，免疫治疗在MPM围手术期具有潜力，但多数研究仍处于早期阶段，需通过大规模随机对照试验验证其疗效及安全性，并结合组织学亚型和生物标志物，推动其个体化治疗策略的发展。

## 2 不可手术切除MPM患者的全身治疗

### 2.1 化疗

对于不可手术切除的MPM患者，通常采用铂类与培美曲塞联合的标准化疗方案，部分患者可继续接受贝伐珠单抗维持治疗^[7]^。对于不能耐受铂类联合治疗的患者，单药化疗（如培美曲塞或长春瑞滨）也是常用选择。尽管这些单药化疗方案在临床试验中显示出一定的治疗活性^[20]^，但与最佳支持治疗（best supportive care, BSC）相比疗效仍需进一步验证。几乎所有MPM患者在一线治疗后最终仍将出现疾病进展，目前只有少数研究表明通过维持化疗可以延长生存期。一项由荷兰18家医院联合进行的II期NVALT19研究^[21]^招募了130例恶性间皮瘤患者，该研究显示，与单纯BSC相比，吉西他滨联合BSC作为维持治疗可显著延长患者的无进展生存期（progression-free survival, PFS），但也伴随3-4级AEs发生比例显著增加（52% *vs* 16%）。组织学类型虽非预测铂类药物疗效的明确指标，但一项回顾性研究^[22]^显示，上皮样型MPM患者在接受一线化疗后的mPFS和mOS分别为4.8和26.7个月，明显优于非上皮型MPM患者的3.6和15.0个月。这一发现提示，上皮样型MPM可能对标准化疗方案具有更高的敏感性，进一步的前瞻性研究仍然需要验证这一差异的临床意义，探索通过个性化治疗策略进一步优化不同组织学类型患者的治疗效果。

化疗反应的个体差异性是当前研究重点，多项研究正探索基于分子生物学的预测模型以推动MPM个体化治疗。Righi等研究^[23]^发现，胸苷酸合酶（thymidylate synthase, TS）蛋白的表达水平可能预测培美曲塞联合铂类药物的疗效。Frischknecht等^[24]^指出，切除修复交叉互补基因1（excision repair cross-complementation group 1, ERCC1）低表达与化疗后较长的未复发持续时间显著相关，而细胞核核糖核苷酸还原酶催化亚基M1（ribonucleotide reductase catalytic subunit M1, RRM1）高表达则可能与接受诱导化疗后行EPP患者的较高生存率相关。尽管标准化疗仍是主要治疗手段，但基于分子标志物的个体化策略为提升疗效提供新方向，需更多大规模前瞻性研究验证其临床价值。

### 2.2 靶向治疗

尽管MPM中高度特异的驱动突变较少见，但某些关键信号通路及基因异常在较高比例患者中存在，这为靶向治疗提供了依据。本研究系统梳理了MPM潜在的治疗靶点，其详细分类及相应靶向药物如图1所示。

**图 1:**
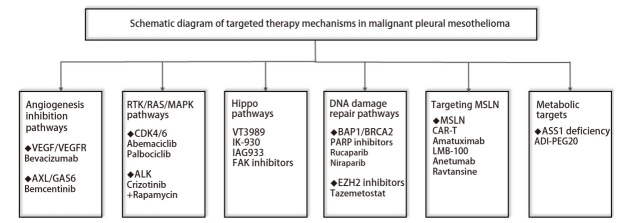
恶性胸膜间皮瘤的靶向治疗机制图示

#### 2.2.1 靶向血管生成抑制

血管内皮生长因子（vascular endothelial growth factor, VEGF）在MPM患者中常呈高表达，因此VEGF信号通路成为治疗的重要靶点^[25]^。III期MAPS研究^[8]^表明，贝伐珠单抗联合培美曲塞和铂类药物治疗可显著延长MPM患者的mOS至18.8个月，mPFS为9.2个月，基于该研究结果，美国临床肿瘤学会（American Society of Clinical Oncology, ASCO）已将其推荐为MPM一线化疗方案之一^[26]^。近期的BEAT-meso试验^[11]^显示贝伐珠单抗加标准化疗（卡铂+培美曲塞）联合阿替利珠单抗治疗可延长PFS（9.2 *vs* 7.6个月），尤其非上皮样型MPM患者OS获益明显（HR=0.51），提示该方案在此类亚型中具有潜力，值得进一步研究验证。除VEGF通路外，受体酪氨酸激酶Axl也被认为是MPM血管生成和免疫逃逸的重要调控因子。II期MiST-3研究^[27]^正在评估Axl抑制剂比美替尼与帕博利珠单抗联合治疗复发性MPM的疗效（88%为上皮样型）。研究结果显示，治疗第12和24周时的疾病控制率（disease control rate, DCR）分别为46.2%和38.5%，达到预设的主要终点，显示该联合疗法在复发性MPM中具有良好的疗效。当前，其分子相关性分析仍在进行中，预计将为精准治疗策略的制定提供更多指导依据。

#### 2.2.2 靶向RTK/RAS/MAPK通路

细胞周期依赖性激酶抑制蛋白2A（cyclin-dependent kinase 2A, *CDKN2A*）基因缺失是MPM中常见的基因改变，该突变导致CDK4/6异常激活，从而促进肿瘤细胞的增殖和存活，因此，CDK4/6抑制剂可能为MPM的潜在治疗策略^[28]^。目前，阿贝西利和帕博西尼等CDK4/6抑制剂已进入临床研究阶段。II期临床试验MiST2^[29]^评估了阿贝西利在既往接受铂类化疗的*p16INK4A*阴性MPM患者中的疗效。结果显示，26例患者中14例（54%）在12周时实现疾病控制（3例PR，11例SD）。此外，*CDKN2A*和甲硫腺苷磷酸化酶（methylthioadenosine phosphorylase, *MTAP*）共同缺失的患者对阿贝西利的反应更佳，提示此类患者可能为阿贝西利治疗的敏感人群。另一种CDK4/6抑制剂帕博西尼在MPM小鼠模型中的研究亦显示出积极结果。相比标准化疗，帕博西尼可更有效缩小肿瘤体积并延长生存期，同时改善肿瘤免疫微环境，增加肿瘤相关巨噬细胞和自然杀伤（natural killer, NK）细胞的聚集，产生抗肿瘤效应^[28,30]^。

#### 2.2.3 靶向Hippo通路

Hippo的上游调控因子Merlin由神经纤维瘤病2型（neurofibromatosis type 2, *NF2*）基因编码，*NF2*失活是该通路最常见的基因变异，约35%-40%的MPM病例中可见，致下游转录因子Yes相关蛋白（Yes-associated protein, YAP）和具有PDZ结合基序的转录共激活子（transcriptional coactivator with PDZ-binding motif, TAZ）活化并驱动转录增强相关结构域蛋白（transcriptional enhanced associate domain, TEAD）依赖性基因转录，促进肿瘤生长^[31]^。针对YAP/TAZ-TEAD复合体的靶向抑制被认为是治疗MPM的潜在策略之一。目前，代表性药物主要包括TEAD棕榈酰化抑制剂VT3989、选择性的TEAD小分子抑制剂IK-930以及直接破坏YAP/TAZ和TEAD相互作用的IAG933。一项I期、开放标签剂量递增临床试验^[32]^表明，VT3989在MPM患者中显示出良好的安全性，未观察到剂量限制性毒性。在42例间皮瘤患者中，6例（14%）获得PR，8周时的临床获益率（PR+SD≥8周）为57%。IK-930和IAG933的I期临床研究正在进行安全性评估，并持续纳入MPM病例。此外，*NF2*缺失增加了MPM对黏附斑激酶（focal adhesion kinase, FAK）信号通路的依赖性，FAK抑制剂GSK2256098在*NF2*缺失的MPM患者中显示出初步的临床益处^[33]^。这些机制的深入研究正在持续推进，有望在未来拓展Hippo通路相关的靶向治疗策略。

#### 2.2.4 靶向DNA损伤修复途径

DNA损伤反应（DNA damage response, DDR）通路是维护基因组稳定性、防止肿瘤发生的核心机制之一。BRCA是DNA双链损伤同源重组修复过程中的重要蛋白，BRCA相关蛋白1（BRCA associated protein 1, BAP1）突变约发生在45%的MPM患者中，BRCA2突变则在2%-8%的MPM患者中存在，这些突变可作为多聚ADP核糖聚合酶（poly ADP-ribose polymerase, PARP）抑制剂治疗的预测生物标志物^[33]^。卢卡帕尼是一种PARP抑制剂，IIa期临床试验（NCT03654833）^[34]^评估了其在26例BAP1缺失的MPM患者中的疗效及安全性。结果显示，12周时的DCR为58%，24周降至23%，所有AEs中，3或4级占比9%，未观察到治疗引起的死亡事件。该研究结果提示卢卡帕尼具有良好的抗肿瘤活性及可控毒性，符合预设的疗效标准。此外，BAP1失活可上调果蝇zeste基因增强子人类同源物2（enhancer of zeste homolog 2, EZH2）表达，进而促进肿瘤生长和侵袭^[35]^。一项II期临床试验（NCT02860286）^[36]^评估了EZH2抑制剂他泽司他在复发或难治性BAP1失活的MPM患者中的疗效。试验结果显示，第12周DCR为54%，提示EZH2抑制可能成为MPM治疗的潜在靶点。

#### 2.2.5 靶向间皮素（mesothelin, MSLN）

MSLN是定位于细胞膜表面的一种糖类蛋白质，在包括MPM在内的多种实体瘤中高度表达。它参与肿瘤的侵袭与恶性转化，并且通常仅在恶性间皮组织中表达，因此被视为MPM治疗的潜在靶点^[37]^。嵌合抗原受体T细胞免疫疗法（chimeric antigen receptor T-cell immunotherapy, CAR-T）靶向肿瘤特异性抗原，增强机体免疫监视并减少肿瘤复发。一项I期临床试验^[38]^评估了MSLN靶向CAR-T细胞胸腔内注射联合帕博利珠单抗的疗效。在25例接受治疗的MPM患者中，有39%在外周血检测中观察到CAR-T细胞持续存在超过100 d，有18例患者接受了CAR-T联合帕博利珠单抗治疗，其mOS为23.9个月。该结果表明，MSLN CAR-T细胞联合程序性死亡受体1（programmed death receptor 1, PD-1）抑制剂可能在MPM治疗中产生协同效果。LMB-100是一种MSLN靶向抗体-毒素偶联物，其与ICIs联合使用时观察到潜在的协同抗肿瘤作用。目前，一项II期研究^[39]^正在评估LMB-100与帕博利珠单抗联合治疗MPM的疗效。

#### 2.2.6 靶向参与新陈代谢的酶

精氨酸琥珀酸合成酶1（argininosuccinate synthetase 1, ASS1）作为尿素循环中精氨酸合成的关键酶，在多种癌症中发挥肿瘤抑制作用。在48%的MPM病例中，ASS1出现缺失，尤其在双相或肉瘤样型MPM中较为常见^[40]^。II期ADAM试验^[40]^评估了聚乙二醇化精氨酸脱亚胺酶（ADI-PEG20）在ASS1缺失型MPM患者中的疗效。结果显示，ADI-PEG20组的mPFS为3.2个月，明显优于BSC（主要涵盖对患者身体、心理、社会和精神层面痛苦状况的评估与治疗）的2.0个月（HR=0.56, P=0.03）。在治疗第4个月时，ADI-PEG20组有52%的患者评估为SD，而BSC组为22%。其中，ASS1缺失程度≥75%且未接受过化疗的患者获益最大。在II/III期ATOMIC-meso试验^[12]^中，ADI-PEG20联合化疗与单独化疗在249例非上皮样型MPM患者进行了疗效比较。结果显示，联合组的mOS为9.3个月，优于单独化疗组的7.7个月，ADI-PEG20联合化疗可能为非上皮型MPM患者提供了一个新的治疗选择。

### 2.3 免疫治疗

MPM被认为具有潜在的免疫原性，石棉肺纤维沉积引起的慢性炎症可能在其发病机制中发挥重要作用。免疫检查点抑制疗法在MPM治疗中的应用日益受到关注。当前，主要靶点包括细胞毒性T细胞相关蛋白-4（cytotoxic T lymphocyte associated protein-4, CTLA-4）、PD-1和程序性死亡配体1（programmed death ligand 1, PD-L1）。尽管免疫疗法可能成为新治疗选择，但其疗效存在异质性。因此，识别潜在获益人群及预测性生物标志物至关重要。

#### 2.3.1 ICIs疗法

替西木单抗是一种CTLA-4抑制抗体，MESOT-TREM-2012研究显示其在双相型或肉瘤样MPM患者中的mOS为15.8个月，较历史数据延长^[41]^。然而，IIb期DETERMINE试验纳入了571例恶性间皮瘤患者，研究结果显示，与安慰剂相比，替西木单抗未能显著延长既往治疗的恶性间皮瘤患者的mOS（7.7 *vs* 7.3个月）^[42]^。尽管替西木单抗为非上皮样型MPM患者可能带来生存获益，但整体疗效有限，未能在大规模试验中显示出显著的生存优势。帕博利珠单抗为一类人源化的抗PD-1单克隆抗体，III期PROMISE-meso试验^[43]^显示，其用于晚期MPM二线治疗的mPFS为2.5个月，低于化疗组的3.4个月，且未发现PD-L1表达与疗效相关。该研究提示帕博利珠单抗在既往治疗失败的MPM患者中的疗效有限。纳武利尤单抗是一种靶向PD-1受体的IgG4单克隆抗体。II期MERIT试验评估了其在既往化疗失败的MPM患者中的疗效，显示客观缓解率（objective response rate, ORR）为29%，mPFS为6.1个月，mOS为17.3个月，毒性可控。且PD-L1表达水平并未造成患者2或3年OS和PFS的显著差异，影响其长期获益。基于该结果，纳武利尤单抗已在日本获批用于铂类化疗后疾病进展的MPM患者^[44,45]^。III期CONFIRM试验^[13]^进一步证实其疗效，在332例复发性恶性间皮瘤患者中纳武利尤单抗组的mPFS和mOS分别为3.0和10.2个月，显著优于安慰剂组（mPFS：1.8个月，mOS：6.9个月），确立了其在二线及以上治疗中的临床价值。

#### 2.3.2 双重ICIs联合

由于单一ICIs疗效的局限性，许多研究聚焦于探索双重ICIs的联合治疗，特别是靶向CTLA-4与PD-1/PD-L1的联合治疗。II期IFCT-1501 MAPS2研究比较了纳武利尤单抗单药治疗与联合伊匹木单抗治疗在培美曲塞和铂类化疗后进展的MPM患者中的疗效。结果显示，单药组和联合组的ORR分别为19%和28%，mOS分别为11.9和15.9个月，联合治疗疗效优于单药治疗。PD-L1高表达（>25%）患者在这两种治疗方案中均表现出更高的应答率，ORR达63%-71%^[46]^。CheckMate 743试验^[9]^是不可切除MPM治疗领域的关键临床研究，促使美国食品药品监督管理局（Food and Drug Administration, FDA）批准了纳武利尤单抗与伊匹木单抗的双免疫联合方案作为不可切除MPM的一线治疗。该试验纳入了605例不可切除MPM患者，随机分为铂类加培美曲塞化疗组和双重ICIs治疗组。结果显示，免疫治疗组的mOS为18.1个月，优于化疗组的14.1个月（HR=0.73, 95%CI: 0.61-0.87）。特别是在非上皮样型患者中，免疫治疗组的mOS为18.1个月，显著优于化疗组的8.8个月（HR=0.48, 95%CI: 0.34-0.69），表明双重ICIs治疗对该亚群患者具有显著的临床获益，提供了有效的一线治疗选择。免疫治疗组在长期生存方面也展现出显著获益，其1及2年生存率均优于化疗组。由于PD-L1表达未作为分层因素，且PD-L1表达<1%组的样本量有限，该研究未能明确评估PD-L1在治疗中的预测作用。

尽管替西木单抗单药在IIb期DETERMINE试验中无生存获益，但研究显示其与度伐利尤单抗的联合治疗仍具潜力。II期NIBIT MESO1研究^[47]^纳入40例恶性间皮瘤患者，联合治疗ORR为28%，mOS为16.6个月，显示出良好抗肿瘤活性，PD-L1表达未能提供预测价值。鉴于样本量较小，研究者强调仍需通过更大规模的研究进一步验证。

#### 2.3.3 ICIs联合化疗

当前，ICIs联合化疗方案在不可切除MPM治疗中展现出潜在的协同增效作用，相关临床研究为其提供了初步依据。在II期DREAM研究^[14]^中，54例既往未经治疗的MPM患者接受了度伐利尤单抗联合顺铂和培美曲塞的化疗方案6个周期，随后进行1年的度伐利尤单抗维持治疗。该研究达到了主要终点，6个月PFS率为57%。研究显示，该联合治疗在各组织学亚型中均显示出一定的抗肿瘤活性，且PD-L1表达水平与PFS未观察到明显相关性。类似的积极结果在PrE0505试验^[15]^中得到了验证，该试验采用了相同的联合治疗方案，观察到的mOS为20.4个月，mPFS为6.7个月。基于这些初步的正面数据，III期DREAM3R研究^[48]^正在进行。JME-001试验^[49]^评估了不可切除MPM患者接受一线顺铂和培美曲塞联合纳武利尤单抗的疗效，结果显示，该方案的ORR为77.8%，mOS为20.8个月，表明纳武利尤单抗联合化疗在不可切除MPM患者中疗效显著。III期IND227研究^[16]^纳入440例晚期MPM患者，评估帕博利珠单抗联合标准化疗的一线治疗效果。结果显示联合组mOS为17.3个月（95%CI: 14.4-21.3），优于化疗组的16.1个月（95%CI: 13.1-18.2），且非上皮样型患者获益更显著（12.3 *vs* 8.2个月）。III期BEAT-meso研究^[11]^评估了在化疗联合贝伐珠单抗治疗弥漫性胸膜间皮瘤中加入PD-L1抑制剂的效果。尽管总体mOS无显著差异（20.5 *vs* 18.1个月，P=0.14），但非上皮样型患者中联合组mOS显著延长（17.9 *vs* 10.0个月，HR=0.50）。

综上所述，当前的临床数据表明，化疗联合ICIs治疗在MPM一线治疗中具有治疗潜力，尤其在非上皮样型患者中可能带来额外生存获益。未来应开展进一步研究，评估生物标志物对MPM患者的预测作用，并探索其对长期生存获益的影响。

## 3 放疗在MPM中的应用

尽管目前不推荐单独使用放疗作为MPM的标准治疗手段，但其作为多学科治疗的一部分发挥着积极作用。姑息性放疗能够缓解胸膜肿瘤进展导致的胸痛、支气管或食管受压、转移引起的头痛、骨痛等症状^[50]^。最近，伴随高精度放射线疗法（intensity-modulated radiation therapy, IMRT）技术的不断进步，为可切除MPM患者实施高剂量半胸放疗提供了可行路径。II期SMART研究^[51]^探索了新辅助短期加速大剂量半胸膜IMRT后行EPP的可行性。该研究中患者的mOS为24.4个月。然而，该方案的围手术期3-4级AEs发生率高达49%，提示该策略需在具备丰富放疗和手术经验的中心谨慎实施。II期IMPRINT研究^[52]^对诱导化疗联合P/D术后半侧胸腔IMRT治疗的安全性进行了评估。结果表明，30%的患者出现了2或3级的放射性肺炎，未见4或5级放疗相关毒性事件。所有可评估患者的OS为23.7个月，可切除肿瘤患者2年OS率为59%。该研究结果支持将该多模式治疗方案作为可行且安全的治疗选择，并已计划开展更大规模的临床试验以进一步验证其疗效。综上所述，在具备高度专业放疗经验的医疗机构中，半侧胸腔放疗可作为MPM患者多学科综合治疗的重要组成部分，前提是严格监测并控制相关毒性。

## 4 新型疗法

树突状细胞（dendritic cell, DC）疫苗可增强抗原呈递与T细胞活化，增强机体对肿瘤的特异性免疫反应，动物实验^[53]^显示其可延长肿瘤模型小鼠生存期，具有抗肿瘤免疫潜力。DENIM研究^[54]^评估了以同种异体MPM细胞裂解物为抗原的DC疫苗（MesoPher）在MPM患者中的维持治疗效果。研究显示，MesoPher治疗组的整体mOS未优于接受BSC组（16.8 *vs* 18.3个月，P=0.62），但美国东部肿瘤协作组（Eastern Cooperative Oncology Group, ECOG）评分为0分的患者PFS显著优于ECOG评分为1分的患者（8.0 *vs* 3.2个月），提示其在体能良好亚组中或具更大治疗潜力。此外，研究者指出，MesoPher与ICIs的联合治疗策略及在特定亚组患者中的应用前景，值得进一步研究和验证。 ONCOS-102是一种经过基因修饰的溶瘤腺病毒，可在肿瘤细胞中复制并重塑免疫微环境，诱导抗肿瘤免疫反应。一项II期探索性临床研究^[55]^结果显示ONCOS-102联合培美曲塞和铂类药物用于治疗MPM患者的安全性良好，未出现因治疗中出现的药物相关AEs导致停药。在首次接受化疗的患者中，ONCOS-102联合治疗组的mOS较单独化疗延长（20.3 *vs* 13.5个月）。免疫分析显示，ONCOS-102可增强肿瘤组织T细胞浸润并上调促炎基因表达。尽管研究显示溶瘤病毒联合化疗在MPM中具有一定疗效与可行性，但受限于样本量，仍需大规模临床试验验证。

## 5 展望与小结

对于大多数MPM患者，内科姑息治疗及症状管理仍为主要治疗手段。尽管现有治疗措施可一定程度延长患者生存期，但总体疗效仍有限。随着对MPM分子生物学特征的深入研究，靶向基因组及表观遗传改变的治疗策略逐步发展，具备临床转化前景。研究重心正逐渐从传统组织学分类转向分子驱动的精准分型。ICIs显著改变了MPM的治疗格局，然而，肉瘤样型MPM的研究相对薄弱，其诊断与治疗策略尚面临诸多挑战。PD-L1表达水平与免疫治疗疗效的相关性亦为研究热点。未来应进一步验证多种生物标志物在诊断及疗效预测中的作用，实现精准分层与个体化治疗。鉴于MPM的高度异质性，免疫联合化疗、靶向治疗等多模式治疗策略有望提高治疗反应率。因此，未来研究应聚焦于优化现有治疗模式，挖掘新的治疗靶点，并探索更个体化的治疗组合，以期为MPM患者提供更有效的治疗选择。
